# How Yeast Antifungal Resistance Gene Analysis Is Essential to Validate Antifungal Susceptibility Testing Systems

**DOI:** 10.3389/fcimb.2022.859439

**Published:** 2022-05-04

**Authors:** Nicolas Pellaton, Dominique Sanglard, Frederic Lamoth, Alix T. Coste

**Affiliations:** ^1^ Institute of Microbiology, University of Lausanne and University Hospital Center, Lausanne, Switzerland; ^2^ Infectious Diseases Service, Department of Medicine, Lausanne University Hospital, Lausanne, Switzerland

**Keywords:** antifungal susceptibility, diagnostic test, resistance genes, CLSI, EUCAST, Sensititre ^TM^ YeastOne ^TM^, MICRONAUT-AM

## Abstract

**Objectives:**

The antifungal susceptibility testing (AFST) of yeast pathogen alerts clinicians about the potential emergence of resistance. In this study, we compared two commercial microdilution AFST methods: Sensititre YeastOne read visually (YO) and MICRONAUT-AM read visually (MN) or spectrophotometrically (MNV), interpreted with Clinical and Laboratory Standards Institute and European Committee on Antimicrobial Susceptibility Testing criteria, respectively.

**Methods:**

Overall, 97 strains from 19 yeast species were measured for nine antifungal drugs including a total of 873 observations. First, the minimal inhibitory concentration (MIC) was compared between YO and MNV, and between MNV and MN, either directly or by assigning them to five susceptibility categories. Those categories were based on the number of MIC dilutions around the breakpoint or epidemiological cut-off reference values (ECOFFs or ECVs). Second, YO and MNV methods were evaluated for their ability to detect the elevation of MICs due to mutation in antifungal resistance genes, thanks to pairs or triplets of isogenic strains isolated from a single patient along a treatment previously analyzed for antifungal resistance gene mutations. Reproducibility measurement was evaluated, thanks to three quality control (QC) strains.

**Results:**

YO and MNV direct MIC comparisons obtained a global agreement of 67%. Performing susceptibility category comparisons, only 22% and 49% of the MICs could be assigned to categories using breakpoints and ECOFFs/ECVs, respectively, and 40% could not be assigned due to the lack of criteria in both consortia. The YO and MN susceptibility categories gave accuracies as low as 50%, revealing the difficulty to implement this method of comparison. In contrast, using the antifungal resistance gene sequences as a gold standard, we demonstrated that both methods (YO and MN) were equally able to detect the acquisition of resistance in the *Candida* strains, even if MN showed a global lower MIC elevation than YO. Finally, no major differences in reproducibility were observed between the three AFST methods.

**Conclusion:**

This study demonstrates the valuable use of both commercial microdilution AFST methods to detect antifungal resistance due to point mutations in antifungal resistance genes. We highlighted the difficulty to conduct conclusive analyses without antifungal gene sequence data as a gold standard. Indeed, MIC comparisons taking into account the consortia criteria of interpretation remain difficult even after the effort of harmonization.

## Introduction

These past years, the global burden of candidiasis remains relatively stable (Lamoth et al., 2018). However, the epidemiology over the last ten years shows a shift from a majority of *Candida albicans* infections toward non-albicans spp. Species such as *C. glabrata* in the northern hemisphere, *C. parapsilosis* in Africa and South America, or *C. tropicalis* in Asia, not to mention *C. auris*, are increasingly reported and are becoming among the most prevalent species in some countries (Lamoth et al., 2018). Such species are known to be naturally less susceptible to antifungal drugs or to rapidly acquire resistance (Silva et al., 2012; Chaabane et al., 2019; Coste et al., 2020; Davari et al., 2020; Chen et al., 2021).

The acquisition of antifungal resistance is essentially due to the acquisition of point mutations in antifungal resistance genes. Azole resistance might be due to point mutations in the *ERG11* gene encoding the azole target, leading to a decrease in drug affinity, the overexpression of the target due to a mutation in Upc2, a transcriptional regulator of *ERG11*, or to an increase in the efflux of the drug. The efflux of azole drugs is due to the overexpression of two types of multidrug transporters, the ATP-binding cassette (ABC) transporters or multifacilitator superfamily (MFS) transporters. ABC transporters are regulated by Tac1 or Pdr1 in *C. albicans* and *C. glabrata*, respectively. MFS transporters are regulated by Mrr1. Mutations in these transcription factors lead to a constitutive expression of these multidrug transporters and to an increase in resistance (Sanglard et al., 2009; Vandeputte et al., 2012; Chaabane et al., 2019). A high level of azole resistance can be achieved by genomic rearrangements, which increase the gene copy number of mutated azole resistance genes (Coste et al., 2007). Echinocandin resistance is essentially due to mutations in the drug target encoded by *FKS1* and/or *FKS2* (Desnos-Ollivier et al., 2008; Vandeputte et al., 2012; Marti-Carrizosa et al., 2015; Chaabane et al., 2019). Polyene resistance has been rarely reported in common *Candida* spp. species but involves a mutation in *ERG* genes, leading to a decrease in ergosterol biosynthesis and altered sterol composition (Young et al., 2003; Hull et al., 2012; Vandeputte et al., 2012; Kannan et al., 2019). All these mechanisms could be cumulated to yield multidrug-resistant strains (Chowdhary et al., 2018; Kannan et al., 2019; Coste et al., 2020). In fact, it is obvious that the detection of antifungal resistance is difficult to implement with molecular tools due to the multitude of genes and mutations involved. Therefore, a phenotypic diagnosis requiring the growth of the microorganism remains the first choice. Several studies have evaluated the potential of mass spectrometry using Matrix Assisted Laser Desorption Ionisation/Time Of Flight (MALDI-ToF) to detect yeast resistance to antifungals, including our study using a machine learning approach (Delavy et al., 2019; Knoll et al., 2021). However, this is still at the conceptual stage. Therefore, a microdilution or E-test measurement of the minimal inhibitory concentration (MIC) of antifungal drugs remains a primary option of choice.

Both CLSI (Clinical and Laboratory Standards Institute) and EUCAST (European Committee on Antimicrobial Susceptibility Testing) propose antifungal susceptibility testing (AFST) protocols and interpretation criteria to detect antifungal resistance or at least a diminution of antifungal susceptibility. However, both reference methods are labor intensive, making them difficult to be implemented in a routine laboratory. In contrast, commercial methods offer a simpler and easy-to- implement alternative (Lombardi et al., 2004; Kritikos et al., 2018; Philips et al., 2021). The Sensititre™ YeastOne™, AFST (Thermo Fisher Scientific, Waltham, MA, United States) follows the CLSI protocol and interpretations, whereas the MICRONAUT-AM Antifungal Agents MIC (MERLIN Gesellschaft fuer mikrobiologische Diagnostika GmbH, Bornheim, Germany) is an adapted protocol of the EUCAST protocol and is interpreted with its criteria. Both are colorimetric broth microdilution tests.

In this study, we aimed to compare the results obtained by both methods with a visual reading of the plates on approximately 90 clinical isolates, essentially comprising *Candida* spp. In addition, we compared the visual reading of the MICRONAUT-AM plates with a reading by a spectrophotometer and software-based result interpretation (MICRONAUT6 software). Some of the isolates were sequentially recovered from a single patient along his/her antifungal treatment and extensively studied for their antifungal resistance mechanisms (Coste et al., 2007; Ferrari et al., 2009; MacCallum et al., 2010; Kannan et al., 2019; Coste et al., 2020; Li et al., 2021).

All along the text, we abbreviated Sensititre™ YeastOne™ as “YO” and MICRONAUT-AM as “MN.” When read visually, the abbreviation used is “MNV” for Micronaut-AM visual.

With both tests being based on different protocols and criteria of interpretation, we assumed that it was not sufficient to compare directly the obtained MIC. It was more indicated to classify these MICs by breakpoint categories or by the epidemiological values (ECV for CLSI and ECOFF for EUCAST) and to evaluate the “category” agreement between the two methods. Facing difficulties with this approach, we then used the antifungal susceptibility genotype of the well-characterized strains as a gold standard to evaluate both tests. This second approach not only clearly allowed an accurate MIC evaluation and therefore a comparison of both tests but also helped with the identification of specific characteristics for each test.

## Material and Methods

### Yeast Strains

All the yeast strains used in this study were described in **Supplementary Table S1**. In summary, 19 yeast species were tested: *C. albicans* (n =22)*, C. auris* (n = 5)*, C. dubliniensis* (n=6)*, C. famata* (n =1)*, C. glabrata* (n=18), *C. guillermondii* (n=3), *C. kefyr* (n=4), *C. krusei* (n=8), *C. lusitaniae* (n=8), *C. metapsilosis* (n=1), *C. nivariensis* (n=1), *C. orthopsilosis* (n=3), *C. parapsilosis* (n=7), *C. tropicalis* (n=5), *Cryptococcus neoformans complex* (n=2), *Cryptococcus laurentii* (n=1), *Saccharomyces cerevisae* (n=1), and *Saprochaete clavata* (n=1), for a total of 97 tested strains.

Two American Type Culture Collection (ATCC) strains (ATCC 22019 C*. parapsilosis* and ATCC 6258 C*. krusei*), and one well-characterized *C. albicans* laboratory strain SC5314 (Gillum et al., 1984) were included as quality controls.

Note that all *C. albicans, glabrata, lusitaniae*, and *auris* strains were already described and analyzed for antifungal susceptibility in previous publications as cited in **Table S1**. The other strains are the isolates selected from our routine during 2019. Antifungal susceptibility testing was not necessarily performed at that time.

### Antifungal Susceptibility Testing

AFST was performed as recommended by the manufacturer for Sensititre™ YeastOne™ and for the MICRONAUT-AM antifungal susceptibility test.

For both methods, using a nephelometer (BioMérieux DENSIMAT), McFarland Standard 0.5 (10^6^ – 5×10^6^ cells/ml) was inoculated in a 2.5 ml 0.85% NaCl sterile tube. Standard inoculation using pure yeast cultures from the SAB agar plate is prepared for a maximum of 24 h beforehand. The same yeast solution is used for the three test plates: YO, MNV, and MN.

For YO, 20 μl of McFarland standard suspension was inoculated in 11 ml of YeastOne inoculum broth (Sensititre YeastOne Broth – Thermo Scientific Y3462). The solution was vortexed for about 10 s and then transferred into a reservoir. An 8-multichannel pipette was used to inoculate 100 μl of culture into a YeastOne microdilutions 96-well plate (Sensititre YeastOne YO10 – Thermo Scientific YO10).

For MN and MNV, 10 μl of McFarland standard suspension was inoculated in 11.5 ml of prepared RPMI-media solutions. Then, 50 versus 100 μl of AST-Indicator (contains reazurin, a component of the AST-Reagent Kit, MERLIN E2-323-001) were added to 11.5 ml of Roswell Park Memorial Institute (RPMI)-media (MICRONAUT-RPMI 1640 + 3-(N-morpholino)propanesulfonic acid (MOPS) + glucose, MERLIN E2-324-020) for MN or MNV, respectively. Only for *C. albicans* and *C. glabrata* strains, 50 μl of methylene blue was added (a component of the AST-Reagent Kit). After vortexing for about 10 s, the solution was transferred into a reservoir and 100 μl of it were dispensed into MN microdilutions 96-well plates (MICRONAUT-AM Antifungal Agents MIC, MERLIN E1-831- 040).

In all three cases, YO, MN, and MNV, plates were sealed and incubated at 35°C for *Candida* strains and 30°C for *Cryptococcus, Saccharomyces*, and *Saprochetae* strains. Readings were performed after 24 and 48 h incubation time.

### MIC Measurements

Both commercial AFSTs contain the same 9 antifungal drugs. Out of the 9 drugs, 3 candins (anidulafungin, caspofungin, micafungin), 4 azoles (fluconazole, itraconazole, posaconazole, and voriconazole), 1 polyene (amphotericin B), and the 5-fluorocytosine (5-FC) were analyzed as follows.

For YO and MNV visual reading at the bottom of the plate, MIC values were assigned, thanks to the reazurin colorimetric marker. A blue-to-pink color shift of the wells indicated significant growth. For fungicides and antifungals such as candins and amphotericin B, MIC values were determined as the first well, which remained blue. For fungistatic azoles, MIC values were determined as the first well that shifted to pink but with a growth at least 50% smaller than the positive control of growth without any drug.

For photometric reading, plates were measured at 492 nm in a Tecan sunrise plate reader and the reading values were analyzed using the MICRONAUT6 software (U8-305-001) provided by MERLIN to determine the MICs (Pfaller et al., 2010).

### Susceptibility Category Assignment

**Figure 1:**
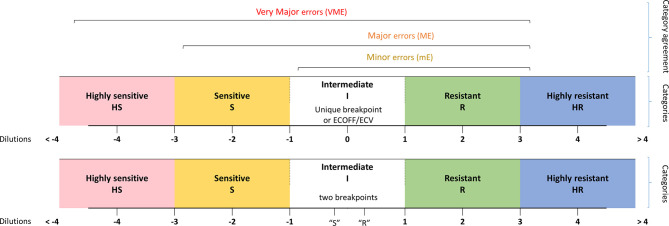
Definition of the MIC categories and agreement levels. Five MIC categories were defined from highly sensitive (HS) to highly resistant (HR), based on dilution differences from a reference MIC value [breakpoint (s) or ECV/ECOFF].

MIC categories were assigned by the number of dilutions between the MIC of isolates and a given epidemiological criteria, either a breakpoint or an ECOFF/ECV when such criteria existed in both consortia. This led us to define five category levels of antifungal susceptibility: highly susceptible (HS), susceptible (S), intermediate (I), resistant (R), and highly resistant (HR). This allows having an idea of the distance to the criteria of interpretation (breakpoint or ECOFF/ECV) and thus an estimated level of MIC. The MIC, which could not be analyzed due to the lack of epidemiological criteria in one and/or the other consortium were qualified as NA (not assigned) (**Figure 1**). Note that when two breakpoints existed, one susceptible and one resistant, we considered that all the MICs between them are categorized in I. In addition, note that for *C. glabrata*, no susceptible breakpoints exist for CLSI. We thus took the SDD (susceptible dose-dependent) breakpoint as a susceptible one.

Comparing the category levels of antifungal susceptibility between YO and MNV, we then determined four categories of agreement: A: agreement (same MIC category), mE: minor error (when YO and MNV have an MIC category with 1-level difference), ME: major error (with 2-level difference), VME: very major error (with 3-level difference).

### Data Analysis

All collected data were analyzed and plotted using R (R Core Team) and RStudio [RStudio Team (2018)]. Notably, the caret package (Max Kuhn (2020); R package version 6.0–86. https://CRAN.R-project.org/package=caret) was used to perform the category agreement, and further statistical analysis: accuracy, kappa score [and sensitivity, specificity, positive predicted value (PPV), negative predicted value (NPV), 95% CI, and Mcnemar’s Test P-value, not shown]. Finally, the following R package was used for data sorting and plotting: ggplot2 reshape, cowplot, mdthemes, gridExtra, and patchwork. All the codes can be consulted at https://gitlab.com/npellato/first_step_code_analysis.

### QC Reproducibility

To evaluate the reproducibility of the three assays, two ATCC strains (ATCC 22019 C*. parapsilosis* and ATCC 6258 C*. krusei*) and one laboratory strain (SC5314 *C. albicans*) were tested ten times on nine different days. On the 9th day, they were measured twice from two different subcultures.

For each strain–antifungal agent combination, the modal MIC was defined. The reproducibility is defined, as described before (Philips et al., 2021), as the percentage of MICs within the modal MIC +/- one dilution, meaning on a 3-dilution range. When the modal MIC consists of two adjacent dilutions, for example, 0.125 and 0.25 mg/L, the reproducibility is defined as the percentage of MICs within a 4-dilution range, so from 0.0625 to 0.5 mg/L for the example given.

## Results

### Comparisons of MIC Values Between Sensititre™ YeastOne™, and MICRONAUT-AM

As a first approach, we compared the MIC raw data obtained with both visual methods YO and MNV. We counted how many MIC were identical or with a one-dilution difference, and considered them as agreeing. MIC with two-dilution differences were considered as minor errors (mEs) and with more than 2 dilutions as major errors (MEs) (**Table 1A**).

**Table 1A:** Comparisons of MIC between YeastOne Sensititre and Micronaut-AM read visually.

	MICs	AB	FC	FZ	VOR	IZ	PZ	MC	AND	CAS	Total comparisons		%	
**ALL**	equal	20	47	47	35	14	9	31	33	30	266	584	**30.47**	**66.9**
	1 dilution	62	26	38	24	52	18	22	35	41	318		**36.43**	
	2 dilutions	14	9	6	20	21	30	22	22	15	159		**18.21**	
	>2 dilutions	1	15	6	18	10	40	22	7	11	130		**14.89**	
***C. albicans***	equal	3	4	11	9	4	0	10	5	8	54	122	27.27	61.62
	1 dilution	13	14	6	5	8	1	7	7	7	68		34.34	
	2 dilutions	6	2	2	4	4	7	3	9	4	41		20.71	
	>2 dilutions	0	2	3	4	6	14	2	1	3	35		4.01	
***C. auris***	equal	0	0	3	0	3	0	1	3	1	11	26	24.44	57.78
	1 dilution	2	3	2	1	0	1	1	2	3	15		33.33	
	2 dilutions	3	2	0	0	2	1	3	0	0	11		24.44	
	>2 dilutions	0	0	0	4	0	3	0	0	1	8		0.92	
***C. dubliniensis***	equal	3	6	1	6	0	4	4	4	2	30	54	55.56	100
	1 dilution	3	0	5	0	6	2	2	2	4	24		44.44	
	2 dilutions	0	0	0	0	0	0	0	0	0	0		0.00	
	>2 dilutions	0	0	0	0	0	0	0	0	0	0		0.00	
***C. glabrata***	equal	5	17	8	2	0	1	9	7	3	52	110	32.10	67.90
	1 dilution	12	0	7	7	9	5	3	7	8	58		35.80	
	2 dilutions	0	0	1	5	7	7	3	3	5	31		19.14	
	>2 dilutions	1	1	2	4	2	5	3	1	2	21		2.41	
***C. krusei***	equal	4	2	4	1	0	0	0	3	2	16	42	22.22	58.33
	1 dilution	3	0	4	2	5	1	1	4	6	26		36.11	
	2 dilutions	1	0	0	3	3	2	2	1	0	12		16.67	
	>2 dilutions	0	6	0	2	0	5	5	0	0	18		2.06	
***C. lusitaniae***	equal	0	3	6	6	0	3	1	2	4	25	54	34.72	75.00
	1 dilution	8	2	1	1	8	1	4	3	1	29		40.28	
	2 dilutions	0	1	1	1	0	2	1	1	0	7		9.72	
	>2 dilutions	0	2	0	0	0	2	2	2	3	11		1.26	
***C. parapsilosis***	equal	0	3	5	4	1	0	0	0	1	14	43	21.88	67.19
	1 dilution	4	3	2	4	6	3	0	2	5	29		45.31	
	2 dilutions	3	1	0	0	0	4	1	4	1	14		21.88	
	>2 dilutions	0	0	0	0	0	0	6	1	0	7		0.80	
***C. tropicalis***	equal	2	4	3	1	0	1	3	3	3	20	36	44.44	80.00
	1 dilution	3	1	1	1	3	1	2	2	2	16		35.56	
	2 dilutions	0	0	0	1	1	2	0	0	0	4		8.89	
	>2 dilutions	0	0	1	2	1	1	0	0	0	5		0.57	
***Cryptococcus sp.***	equal	0	0	0	0	0	0	3	3	0	6	14	22.22	51.85
	1 dilution	3	1	2	0	1	0	0	0	1	8		29.63	
	2 dilutions	0	1	1	2	2	0	0	0	2	8		29.63	
	>2 dilutions	0	1	0	1	0	3	0	0	0	5		0.57	
**Other yeasts**	equal	3	8	6	6	6	0	0	3	6	38	84	28.36	62.69
	1 dilution	11	2	8	4	6	3	2	6	4	46		34.33	
	2 dilutions	1	2	1	4	2	5	9	4	3	31		23.13	
	>2 dilutions	0	3	0	1	0	7	4	2	2	19		2.18	

Considering that one dilution difference is not significant, a 66.9% (584/873 MICs) global agreement is obtained between the two methods (**Table 1A**). The best agreements is observed with amphotericin B (only 1 ME). The lowest agreement is obtained with the pozaconazole. 40 ME were observed representing more than 40% of the comparisons, with systematic lower values with MNV. Then, considering that mE are acceptable, we included the 159 MICs with a 2-dilution difference between the two methods in the comparable MIC. We thus obtained an acceptable agreement of 85.11% (743/873 MICs).

For *C. albicans*, and *C. glabrata*, the global agreement is 61.6%, and 67.9%, respectively. *C. dubliniensis* obtained a perfect agreement of 100% of identical MIC obtained with the two methods. *C. auris, C. krusei*, and the fours Cryptococci are the only three “species” with agreement lower than 60% (57.8% and 58.3%, and 51.8%, respectively).

This indicates that globally, only 2/3 of the MIC are directly comparable between the two methods. An additional 20% shows an mE between the two methods and could be considered as acceptable. However, their interpretations will be different between the two methods as they are following the criteria of the two different consortium. With interpretation being the most valuable result used by non-expert clinicians, we then analyze the interpretation agreement between the two methods.

### Comparisons Between Sensititre™ YeastOne™, and MICRONAUT-AM by Category Agreement

Thus, considering that it was not sufficient to compare directly the MICs between the two kits because of the difference in MIC interpretation, we assigned each MIC to one of the five category levels of antifungal susceptibility (I, S, HS, R, and HR), to get a relative estimate of the MIC in relation to the interpretation criteria, as described in the *Material and Methods* and then compared these categories.

First, we defined which species–antifungal combinations MICs could be analyzed using breakpoint or using ECOFF/ECV (**Table S2**). In the end, 873 isolate–antifungal combinations (97 strains × 9 antifungal drugs) were compared between the three AFST methods.

We next determined that using breakpoints as epidemiological criteria, 6 out of 19 species of yeast (*C. albicans, C. glabrata, C. krusei, C. parapsilosis, C. tropicalis*, and *C. orthopsilosis*) could be analyzed for their susceptibility to 5 out of 9 antifungal drugs (anidulafungin, caspofungin, micafungin, fluconazole, and voriconazole). Thus, only 193/873 (22.1%) combinations could be compared between the 3 AFST methods using breakpoints as criteria. Among them, 73.6% concerned *C. albicans* and *C. glabrata* (**Table S2**).

Using ECOFF/ECV as epidemiological criteria, 11 out of 19 species of yeasts (C. albicans, C. glabrata, C. lusitaniae, C. dubliniensis, C. kefyr, C. guillermondii, C. auris, C. krusei, C. parapsilosis, C. tropicalis, and Cryptococcus neoformans complex) could be analyzed for their susceptibility to 7 out of 9 antifungal drugs (amphotericin B, anidulafungin, micafungin, fluconazole, itraconazole, voriconazole, and posaconazole). Thus, 433/873 (49.6%) combinations could be compared between the 3 AFST methods using ECV/ECOFF as criteria. Among them, 54.5% concern C. albicans and C. glabrata (**Table S2**). Note that some combinations already analyzed using breakpoints could also be analyzed using ECV/ECOFF.

Lastly, 352/873 (40.3%) isolates–antifungals combinations could not be analyzed by category agreement as no epidemiological criteria could be used for comparison (**Table S2**). *C. albicans* and *C. glabrata* comprised 30.1% of the combination that could not be compared due to the lack of any criteria for flucytosine in both consortia for any species and to the lack of criteria for caspofungin, itraconazole, and posaconazole in one of the two consortia for *C. albicans* (**Table S2**).

**Figure 2:**
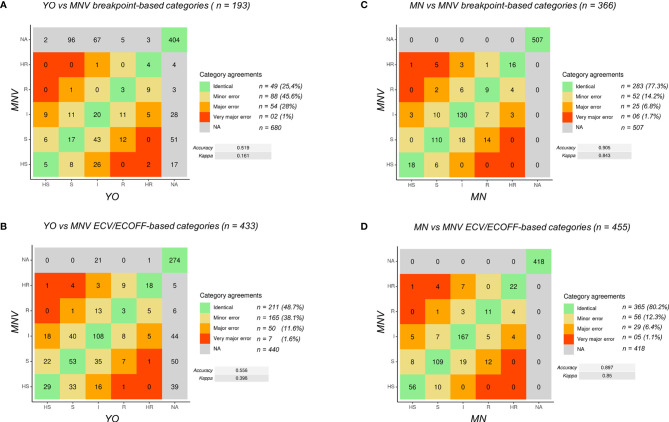
Confusion matrix of MIC categories for all the data. **(A)** Comparison of YO versus MNV using breakpoints. **(B)** Comparison of YO versus MNV using ECV/ECOFF. **(C)** Comparison of MNV versus MN using breakpoints. **(D)** Comparison of MNV versus MN using ECV/ECOFF. VME, very major error (in red); ME, major error (in orange); mE, minor error (in yellow), NA, no analysis (in gray); no errors (in green). For each category of discrepancies, the number of event is indicated (n = xx) with the percent of possible comparisons it represents. Accuracy between the two methods and kappa score are indicated on the right side of the matrix.

Using an R pipeline, we then attributed, when possible, a category to the measured MIC and compared the category obtained between two selected methods (**Figure 2**). The confusion matrixes were obtained with statistical values for MIC category comparisons using breakpoint (**Figures 2A, C**) and ECV/ECOFF (**Figures 2B, D**).

First, to validate the MIC categorization approach, we compared the data obtained with MN and MNV (**Figures 2C, D**) using the two types of criteria breakpoints and ECV/ECOFF. The accuracy was equal to 0.905 (kappa 0.843) using breakpoints and 0.897 (kappa 0.85) using ECV/ECOFF. Looking at the raw data, 90% of the raw MIC values were similar (equal or having a difference of one dilution) between MN and MNV protocols (**Table 1B**), confirming that our categorization approach was valid.

**Table 1B:** Comparisons of MIC between Micronaut-AM read visually and spectrophometrically.

	MICs	AB	FC	FZ	VOR	IZ	PZ	MC	AND	CAS	Total comparisons	%	
**ALL**	equal	78	70	58	67	73	68	59	27	61	561	**64.26**	**89.35**
	1 dilution	18	19	32	24	14	18	29	35	30	219	**25.09**	
	>1 dilution	1	8	7	6	10	11	9	**35**	6	93	**10.65**	
***C. albicans***	equal	15	19	14	19	19	19	18	12	12	147	74.24	**93.94**
	1 dilution	6	2	7	2	2	2	3	8	7	39	19.70	
	>1 dilution	1	1	1	1	1	1	1	2	3	12	6.06	
***C. auris***	equal	4	5	2	3	3	4	1	0	4	26	57.78	84.45
	1 dilution	1	0	3	2	0	1	3	1	1	12	26.67	
	>1 dilution	0	0	0	0	2	0	1	**4**	0	7	15.56	
***C. dubliniensis***	equal	5	6	6	6	6	6	2	0	2	39	72.22	90.74
	1 dilution	1	0	0	0	0	0	4	1	4	10	18.52	
	>1 dilution	0	0	0	0	0	0	0	**5**	0	5	9.26	
***C. glabrata***	equal	16	4	15	16	9	14	14	1	9	98	60.49	92.6
	1 dilution	2	13	2	2	4	4	2	15	8	52	32.10	
	>1 dilution	0	1	1	0	5	0	2	2	1	12	7.41	
***C. krusei***	equal	7	3	2	1	5	0	5	0	8	31	43.06	77.78
	1 dilution	1	2	6	7	3	5	1	0	0	25	34.72	
	>1 dilution	0	3	0	0	0	3	2	**8**	0	16	22.22	
***C. lusitaniae***	equal	7	5	4	6	6	8	4	2	6	48	66.67	88.9
	1 dilution	1	2	3	2	2	0	4	1	1	16	22.22	
	>1 dilution	0	1	1	0	0	0	0	**5**	1	8	11.11	
***C. parapsilosis***	equal	4	7	4	7	7	7	5	3	6	50	79.37	98.42
	1 dilution	3	0	3	0	0	0	2	3	1	12	19.05	
	>1 dilution	0	0	0	0	0	0	0	1	0	1	1.59	
***C. tropicalis***	equal	5	4	0	1	3	0	0	0	2	15	33.33	48.9
	1 dilution	0	0	1	0	0	0	3	0	3	7	15.56	
	>1 dilution	0	1	4	4	2	5	2	**5**	0	23	51.11	
***Cryptococcus sp.***	equal	3	3	1	1	2	0	3	3	3	19	67.8	92.8
	1 dilution	0	0	2	2	1	2	0	0	0	7	25	
	>1 dilution	0	1	0	0	0	1	0	0	0	2	7.2	
**Other yeasts**	equal	12	14	10	7	13	10	7	6	9	88	65.18	94.1
	1 dilution	3	0	5	7	2	4	7	6	5	39	28.88	
	>1 dilution	0	1	0	1	0	1	1	3	1	8	5.9	

Then, we compared the categories obtained for YO and MNV, both methods being read visually. Globally, the accuracy was as low as 0.519 (kappa 0.161) between the two kits read visually using breakpoints and 0.556 (kappa 0.398) using ECV/ECOFF. Forty-nine out of 193 comparisons using breakpoints were identical, and 88 were categorized as mE (representing 45.6% of the comparisons), 54 (28%) as ME, and two (1%) as VME. Considering ECV/ECOFF as the criteria for comparisons, 211 out of 433 comparisons were identical, representing 48.7% of the comparisons. One hundred sixty-five (38.1%) were considered as mE, 50 (11.6%) as ME, and seven (1.6%) as VME. Even if the global accuracies of the two types of comparisons were low, when considering the identical comparisons and those with an mE as “acceptable,” we obtained 71% of “acceptable agreement” between both methods using breakpoint as criteria, and 86.8% of using ECV/ECOFF. Looking in detail at the errors using breakpoints as the criteria of comparisons, we observed that 22 out of the 54 VMEs and MEs are obtained with anidulafungin and 24 out of 54 with micafungin (**Table S3**). The majority of them concerned *C. albicans* (**Table S3**; **Figure S1**). Errors using breakpoints as the criteria of comparisons included essentially *C. glabrata* with 27 out of 65 of the VME and ME, the majority of them concerning anidulafungin, itraconazole, and voriconazole (**Table S3**; **Figure S1**).

### Comparisons Between Sensititre™ YeastOne™, and MICRONAUT-AM Based on the Characterization of Antifungal Resistance Genes

One of the main properties of a susceptibility test is to reveal the presence of molecular changes (mutations or aneuploidy) related to antifungal resistance by raising the MIC. We thus addressed the respective ability of both tests to at least detect the diminution of antifungal susceptibility in strains harboring mutations and/or aneuploidy involving resistance genes, by an increase of the measured MIC.

As stated above, pairs or triplets of strains were sampled from the same patient along his/her antifungal treatment, and which became resistant to one or several antifungals. Those strains were previously analyzed in our laboratory to determine the molecular basis of the antifungal resistance (Coste et al., 2007; Ferrari et al., 2009; MacCallum et al., 2010; Kannan et al., 2019; Coste et al., 2020). This included 20 C*. albicans* strains isolated from 11 different patients, 17 out of 18 C*. glabrata* strains isolated from 9 patients, and 5 C*. lusitaniae* strains isolated from a single patient.

**Figure 3:**
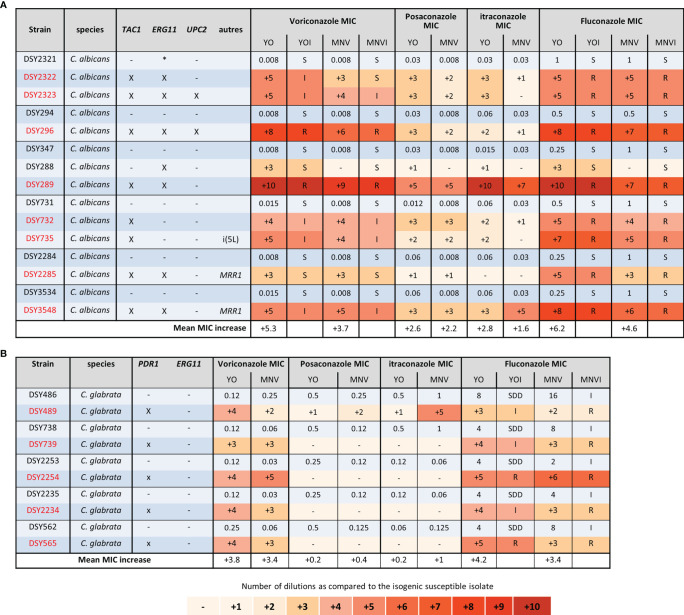
Azole MIC increases of *C. albicans* and *C. glabrata *resistant strains as compared to matched susceptible ones for YO and MNV. **(A)** *C. albicans* matched strains. **(B)** *C. glabrata* matched strains. For all susceptible isolates, the MIC values are indicated in micrograms per millilliter of drugs, whereas for the matched resistant isolates, the respective number of azole dilutions to reach the MIC is indicated. When possible, the interpretation of the MIC, relative to existing breakpoints, is indicated in the YOI (for YO) and MNVI (for Micronaut-AM) columns.

We first analysed the groups of C. albicans and glabrata strains isolated, before the 2000 years, and thus the release of candins on the market, from patients exposed to azoles treatments (**Figure 3**). In each *C. albicans* group of strains, mutations in *TAC1*, *ERG11*, *MRR1*, and *UPC2* genes were characterized in the azole-resistant isolates (Coste et al., 2007; MacCallum et al., 2010). We could clearly observe that both systems displayed increased MICs for the strains carrying mutations in those genes, except for the DSY288, which carried a mutation in *ERG11* only. In this last case, the voriconazole and fluconazole MIC increased by 3 dilutions compared to the initial susceptible isolated with the YO methods but not with the MNV method (**Figure 3A**). However, both methods, measuring or not an increased MIC, interpret it as susceptible (**Figure 3A**). We could also observe that both methods detected for all the strains, except DSY289, there are higher MIC elevations for voriconazole (with a mean increase of 5.3 and 3.7 with YO and MNV, respectively) and fluconazole (mean MIC increase of 6.2 and 4.6 with YO and MNV, respectively) than for posaconazole (mean MIC increase of 2.6 and 2.2 with YO and MNV, respectively) and itraconazole (mean MIC increase of 2.8 and 1.6 with YO and MNV, respectively) (**Figure 3A**). All strains carrying a mutation in *PDR1* in the *C. glabrata* strain pairs (Ferrari et al., 2009) displayed increased MIC with both methods for voriconazole and fluconazole as compared to the matched susceptible isolates but not for posaconazole nor itraconazole (**Figure 3B**). We could also notice that globally, the YO method displayed increases with a higher magnitude of azole MIC than the MNV method (a global *C. albicans* azole MIC mean increase of 4.2 versus 3 with YO and MNV, respectively, and a global *C. glabrata* azole MIC mean increase of 4 versus 3.4 with YO and MNV, respectively) (**Figure 3**).

**Figure 4:**
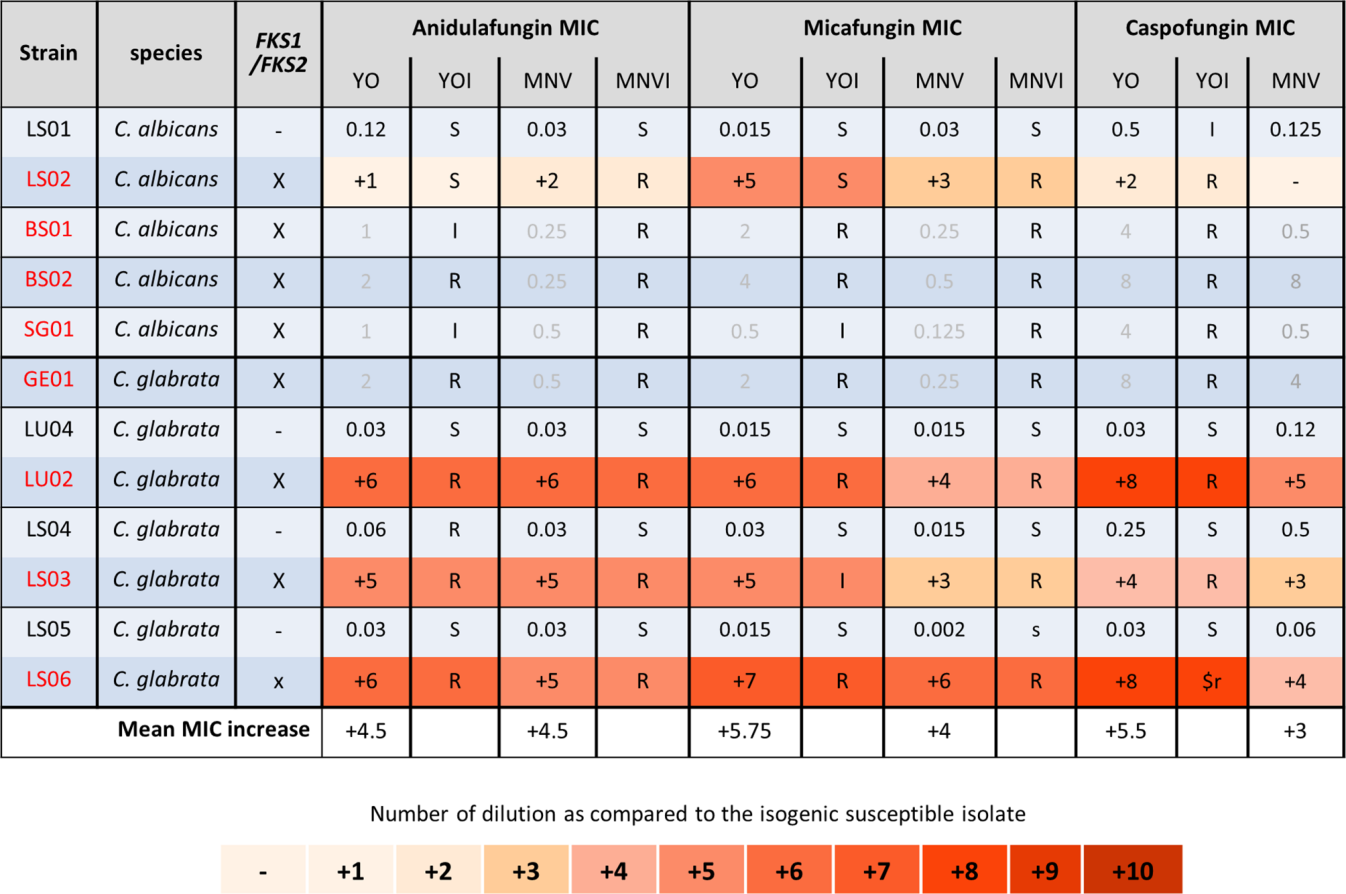
Candin MIC increases of *C. albicans* and *C*. *glabrata* resistant strains as compared to matched susceptible ones for YO and MNV. **(A)** *C. albicans* matched strains. **(B)** *C. glabrata* matched strains. For all susceptible isolates, the MIC values are indicated in micrograms per millilliter of drugs, whereas for the matched resistant isolates, the respective number of azole dilutions to reach the MIC is indicated. When possible, the interpretation of the MIC, relative to existing breakpoints, is indicated in the YOI (for YO) and MNVI (for Micronaut-AM) columns.

We next analyzed the *C. albicans* and *C. glabrata* strains of patients treated with candins for at least 2 weeks (**Figure 4**) (Coste et al., 2020). All the strains carrying mutations in *FKS1* for *C. albicans*, or in *FKS1* or *FKS2* for *C. glabrata*, displayed an elevated MIC for the three candins with both methods (**Figure 4**). For four of the *C. albicans* strains carrying an *FKS1* mutation, we did not obtain a corresponding susceptible isolate. We thus could not calculate candins’ MIC increase. However, both systems gave anidulafungin and micafungin MICs interpreted as resistant or at least intermediate with the YO method. No interpretation of caspofungin MIC could be given for MNV as no breakpoint exists in EUCAST. YO methods gave caspofugin-resistant MICs (**Figure 4**). Once again, the YO method displayed higher increases of MIC than the MNV method.

**Figure 5:**
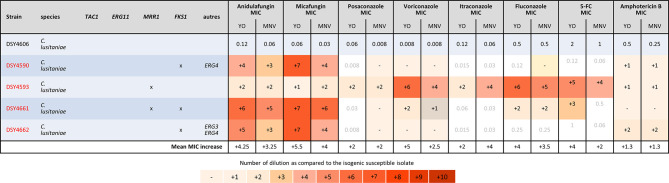
MIC increases of *C. lusitaniae* resistant strains as compared to the matched susceptible one for YO and MNV. The MIC values are indicated for the susceptible isolate in micrograms per millilliter of drugs, whereas for the matched resistant isolates, the respective number of azole dilutions to reach the MIC is indicated. As none of the drug has breakpoint interpretation in both consortia for *C. lusitaniae*, we do not indicate any interpretation in this table.

Finally, we analyzed the *C. lusitaniae* sequential isolates of a single patient (**Figure 5**). Both methods were able to detect the increase of anidulafungin and micafungin MICs due to *FKS1* mutations and amphotericin B MIC increase due to *ERG4* and/or *ERG3* mutations. We observed an increase of two dilutions of amphotericin B MIC when both genes were mutated versus one dilution when only *ERG4* was mutated with both methods. The effect of the *MRR1* mutation could be detected by both methods for all azoles and 5-FC in the strain DSY4593. However, in the strain DSY4661, which carried *MRR1* and *FKS1* mutations, only voriconazole and fluconazole MIC increase was detected by both methods. None of the methods detects an MIC increase for posaconazole nor itraconazole (this strain was measured four times by both methods obtaining each time the same results, data not shown). In this strain, only the YO methods detect a 5-FC MIC increase (**Figure 5**).

### Agreement Between Visual and Spectrophotometric Interpretation of the MICRONAUT-AM

One of the aims of this study was to implement eventually an automatic reading and interpretation of the AFST results to avoid subjective interpretation and transcription error in our laboratory informatics system (LIS). We thus aimed to evaluate the liability of the spectrophotometric reading of the MN method as compared to the MNV method. For this purpose, we compared the MIC raw data obtained with both methods. We counted how many MICs were identical, with one dilution or more than one dilution difference with the MN and MNV methods (**Table 1B**).

Considering that one dilution difference is not significant, an 89.35% (780/873 MICs) global agreement was obtained between the two methods. The best agreement was observed with amphotericin B (99% agreement) (**Table 1B**). The lowest agreement was obtained with anidulafungin (64% agreement), with around one-third of the MICs showing more than one dilution difference between the two methods (**Table 1**). We observed that anidulafungin and micafungin discordances were due to the lower MIC read by the spectrophotometer. However, it seems to be the opposite for the discordances observed for azoles and 5-FC (see All_data_MIC.xls in the GitHub depository as mentioned in *Material and Methods*). We might thus underestimate the echinocandin’s susceptibility, especially anidulafungin, using the spectrophotometer dedicated for Micronaut-AM plate reading.

For *C. albicans*, and *C. glabrata*, the global agreement was 93.94% and 92.6%, respectively. *C. parapsilosis* obtained an almost perfect agreement of 98.42%. *C. tropicalis* and *C. krusei* were the only two species with an agreement lower than 80% (48.9% and 77.78%, respectively).

### Quality Control Reproducibility Analysis

In order to evaluate the reproducibility of the tests, three strains, including *C parapsilosis* ATCC 22019, *C. krusei* ATCC 6258, and *C. albicans* SC5314, were measured ten times on 9 different days, with two independent measurements on the last day. There was a relatively high reproducibility between 90% and 100% of the majority of the results in the Mode +/- 1 dilution range (**Table 2**). The YO tests showed increased variability for itraconazole with 70% and 50% of reproducibility for *C. parapsilosis* and *C. albicans*, respectively. Seventy percent of reproducibility was also obtained for posaconazole with *C. krusei*. Average values were obtained with the MNV or MN tests. The lowest reproducibility of 80% was obtained with anidulafungin and *C. parapsilosis*.

**Table 2:** YO, MNV, and MN reproducibility.

QC strain	Antifungal drug	CLSI Modal MIC (mg/L)	Philips et al. (2021) Modal MIC (mg/L)	YO Modal MIC (mg/L)	% in the +/-1 dilution limit	EUCAST Modal MIC (mg/L)	Philips et al. (2021) Modal MIC (mg/L)	MNV modal MIC (mg/L)	% in the +/-1 dilution limit	MN modal MIC (mg/L)	% in the +/-1 dilution limit
**ATCC 6258 *C. krusei***	Anidulafungin	0.06	0.12	0.03	100	0.03	0.03	0.125	90	0.015	100
	Micafungin	0.25	0.12-0.25	0.12	100	0.06	0.06	0.03	90	0.015	100
	Caspofungin	0.5	0.5	0.25	100	NA	0.25	0.25	100	0.12	100
	5-FC	8	16	8	90	2	8	1	100	1	100
	Posaconazole	0.25	0.25	0.25	70	0.03	0.03	0.015	90	0.015	90
	Voriconazole	0.25	0.25	0.25	90	0.06-0.12	0.03	0.03	90	0.06	90
	Itraconazole	0.5	0.25	0.12-0.25	90	0.06	0.03	0.03	90	0.03	90
	Fluconazole	16		32	100	32		16	100	16	100
	Amphotericin B	1		0.5-1	100	0.25-0.5		0.5	100	0.5	90
**ATCC 22019 *C. parapsilosis***	Anidulafungin	1	1	1	100	0.5	0.25	0.25	100	0.12	80
	Micafungin	1	1	1	100	1	0.12	0.25	90	0.12	90
	Caspofungin	0.5	0.5	0.5	100	NA	0.12-0.25	0.25	100	0.25	100
	5-FC	0.12	0.5	0.25	90	0.25	0.06	0.06	100	0.06	100
	Posaconazole	0.12	0.06	0.06	90	0.03	0.008	0.008	90	0.008	90
	Voriconazole	0.06	0.015	0.03	100	0.03	0.008	0.008	90	0.008	90
	Itraconazole	0.25	0.12	0.12	70	0.06	0.03	0.03	100	0.03	90
	Fluconazole	2	1	2	100	1	1	1	100	1	90
	Amphotericin B	0.5	0.5	0.5-1	100	0.25-0.5	0.25	0.25	100	0.25-0.5	100
**SC5314 *C. albicans***	Anidulafungin	NA		0.12	80	NA		0.03-0.06	100	0.03	100
	Micafungin			0.015	100			0.03	100	0.015	100
	Caspofungin			0.06	100			0.12	100	0.06	100
	5-FC			0.12	100			0.006	100	0.06	100
	Posaconazole			0.015	100			0.008	100	0.008	100
	Voriconazole			0.008	80			0.008	100	0.008	100
	Itraconazole			0.015	50			0.03	100	0.03	100
	Fluconazole			0.25	90			0.5	100	0.5	100
	Amphotericin B			1	100			0.25	100	0.25	100

Each strain was measured 10 times on nine different days. On the 9th day, strains were measured twice from two different subcultures.NA, Not available.

We could also observe that our modal MICs obtained with YO were equivalent at a dilution close to those described by CLSI for *C. parapsilosis* and *C. krusei*. In contrast, our MODAL MICs obtained with MN or MNV were more than 1 dilution apart for anidulafungin with *C. krusei* and posaconazole, voriconazole, and candins with *C. parapsilosis* than those described by EUCAST. These data were close to those reported by Philips et al. (2021).

## Discussion

In this study, we used two original approaches to compare two commercial microdilution AFST methods, one based on CLSI criteria and the other one on EUCAST criteria. The first approach based on the categorization of the MIC allows a comparison of the ability of both methods to assign a similar degree of susceptibility or resistance to antifungals. The greatest difficulty was to define arbitrarily the dilution intervals of the different categories. As discussed below, the level of MIC elevation for the same resistance mutation is different for each method. Therefore, although both methods detect the occurrence of resistance, our approach may assign different categories, resulting in an mE or even ME between the two methods. This probably explains why we obtained relatively low overall accuracy. In addition, our analysis is based on only 97 strains essentially composed of *C. albicans* and *C. glabrata* (around 50% of the strains). However, in Europe, those two species are the most frequently present in candidiasis (Lamoth et al., 2018).

In addition, due to the lack of criteria for some species–antifungal combinations, almost one-third of our observations could not be analyzed. Indeed, non-*albicans* and non-*glabrata* species lack breakpoints in both consortia such as *C. kefyr*, *C. meta*- and *ortho-psilosis*, *C. famata*, *C. nivariensis*, and *Cryptococcus* spp. Flucytosine MIC could not be compared due to the lack of criteria in both consortia, caspofungin due to the lack of criteria in EUCAST, and itraconazole and posaconazole due to the lack of criteria in CLSI. Amphotericin B could be compared only based on ECV/ECOFF, since CLSI does not provide breakpoints for this drug. Our analysis also highlighted the discrepancies between the two methods with specific drugs such as anidulafungin and micafungin as described by Philips et al. (2021), who identified 21% of the MEs attributable to anidulafungin when comparing YO and MNV raw MICs. In contrast, other studies, comparing E-test with EUCAST criteria and YO, revealed a very good agreement between the two methods (Aigner et al., 2017). In addition, our results tend to show higher MIC, and therefore different categories, with CLSI than EUCAST microdilutions for echinocandins in contrast to previous studies using standard microdilutions methods (Pfaller et al., 2010; Pfaller et al., 2014; Meletiadis et al., 2016). We wonder if this difference was attributable to the adaptation of the two original methods to commercial ones. All these observations lead to the conclusion that despite efforts of standardization (Pfaller et al., 2010; Pfaller et al., 2014; Chowdhary et al., 2015; Beredaki et al., 2020), the two consortia still have to make efforts to provide criteria and protocols that are more similar to simplify AFST among all laboratories. Some may claim that differences will remain due to the difference of sampling and thus epidemiology.

Our second approach permitted to verify that both methods detected efficiently the acquisition of mutations involved in antifungal resistance. We fully agree that point mutations are not the only source of reduced susceptibility to antifungal agents, but to our knowledge, resistance or reduced susceptibility to antifungal agents that is not related to a point mutation is more related to transient resistance due to stress (sensu lato) or to a tolerance mechanism (Berman and Krysan, 2020). In the latter two situations, *in vitro* no antifungal susceptibility test is efficiently able to detect and quantify them. However, a direct comparison of the two tests appeared to be irrelevant, probably due to different protocols and, above all, different interpretation criteria. We therefore chose this approach to, at least, evaluate the sensibility of the two tests to reveal the effect of point mutations in resistance genes described to impact on antifungal susceptibility. We observed that in strains harboring mutations, the elevation of MIC was more pronounced with YO using CLSI criteria than with MNV, using EUCAST criteria. Indeed, previous studies already observed that CLSI MIC has a tendency to be higher than the EUCAST for more drugs and species (Pfaller et al., 2014; Philips et al., 2021). (Philips et al., 2021), and in contrast to (Pfaller et al., 2014), we observed that this difference was also true for caspofungin at least for the *C. glabrata* isolates analyzed. Like (Philips et al., 2021), but on very few strains showing the elevation of itraconazole MIC increase, we also observed a slightly higher increase with MNV than YO.

However, for both methods, we could observe that the MIC of azoles with long side-chains, such as itraconazole and posaconazole, was much lower than the MIC of azoles with short side-chains such as fluconazole and voriconazole. We did not observe any increase in MICs when *PDR1* was mutated in *C. glabrata* (except for DSY489) for itraconazole and posaconazole. Indeed, this difference in MIC elevation might be due to the difference of the structure of the long (itraconazole and posaconazole) versus short (fluconazole and voriconazole) side-chain azoles, which impacts their interaction with not only their target but also different transporters as already described (MacCallum et al., 2010; Shafiei et al., 2020; Shi et al., 2020; Borgeat et al., 2021). In addition, we observed as mentioned before (MacCallum et al., 2010) that the presence of only one mechanism of resistance, *ERG11* or *TAC1* mutations, in *C. albicans* leads to moderate azoles’ MIC increase, which might be missed by the MNV methods as observed for DSY288 (**Figure 3**). This is probably due to the global lower MIC increase observed with EUCAST based-methods as mentioned above. We also observed that even if strains carried the same type of azole resistance mechanisms, the MIC increase degree is not similar but detected equally with the two methods. This difference in MIC increase degree is probably due to the different gain-of-function mutations in the resistance gene, which affect differently the properties of the encoded proteins (Coste et al., 2007; Morio et al., 2010; Liu and Myers, 2017).

With regard to the reproducibility of the three methods, MN, MNV, and YO, we did not notice major differences through the repetitions of the three CQ strains (*C. parapsilosis* ATCC 22019, *C. krusei* ATCC 6258, and *C. albicans* SC5314). We noticed that the measurements performed on the fourth day showed MIC more than two dilutions different from the modal MIC for almost all the drugs with the three methods and the two ATCC strains. Eliminating these data, we obtained much better results with almost 100% of the measurements in the +/- 1 dilution limit to the modal MIC. However, we observed, like Philips et al. (2021), that the modal MICs were slightly different for *C. krusei* ATCC 6258 with the three methods but clearly lower for *C. parapsilosis* ATCC 22019 in particular echinocandins and the MICRONAUT-AM system. This important difference must be taken into account during the quality control of the MICRONAUT-AM system. We believe that these quality controls should be based on *C. krusei*.

Lastly, we did not observe major differences between the visual (subjective) and spectrophotometric (automatic) MIC of the MICRONAUT-AM system. In addition, a comparison of the raw MIC values showed 90% agreement between the two methods for all the strains. This is very interesting, as we would have expected differences due to the trailing phenomenon, especially in *C. albicans* and with fluconazole. However, the difference between the two methods for this parameter is above 95% with only one discordance on 22 analyzed strains (**Table 1B**). We consider that the automatic reading of MICs is an added value in order to limit subjective interpretations and transcription errors, as long as the reading system is directly connected to the LIS.

In conclusion, this study clearly demonstrates that both methods, YO and MNV, are valuable, each with limitations and advantages, which have to be taken in consideration before implementation in routine analysis and when interpreting AFST results.

## Data Availability Statement

The datasets presented in this study can be found in online repositories. The names of the repository/repositories and accession number(s) can be found below: https://gitlab.com/npellato/first_step_code_analysis.

## Author Contributions

AC, NP, DS, and FL contributed to the conception and design of the study. NP and AC organized the database. NP performed the statistical analysis. AC and NP wrote the first draft of the manuscript. All authors contributed to manuscript revision, read, and approved the submitted version.

## Funding

Open access funding provided by University of Lausanne.

## Conflict of Interest

The authors declare that the research was conducted in the absence of any commercial or financial relationships that could be construed as a potential conflict of interest.

All MICRONAUT materials have been provided by MERLIN Gesellschaft fuer mikrobiologische Diagnostika mbH as stated in our research agreement contract.

## Publisher’s Note

All claims expressed in this article are solely those of the authors and do not necessarily represent those of their affiliated organizations, or those of the publisher, the editors and the reviewers. Any product that may be evaluated in this article, or claim that may be made by its manufacturer, is not guaranteed or endorsed by the publisher.

## Supplementary Material

Supplementary Figure 1 Confusion matrix of MIC categories for *C. albicans* and *C. glabrata*. **(A)** Comparison of YO versus MNV using breakpoints for *C. albicans*, **(B)** Comparison of YO versus MNV using ECV/ECOFF for *C. albicans*, **(C)** Comparison of YO versus MNV using breakpoints for *C. glabrata*, **(D)** Comparison of YO versus MNV using ECV/ECOFF for *C. glabrata*. VME: Very Major Error in red, ME: Major Error in orange, mE: Minor error in yellow, no errors in green and no analysis (NA) in grey. For each category of discrepancies, the number of event is indicated (n = xx) with the percent of possible comparisons it represents.

Supplementary Table 1 List of the strains used in this study.

Supplementary Table 2 Summary of the breakpoints and ECV/ECOFF available in CLSI and EUCAST consortia.

Supplementary Table 3 YO vs MNV comparisons discrepancies.
