# Supplementary material for: How Yeast Antifungal Resistance Gene Analysis Is Essential to Validate Antifungal Susceptibility Testing Systems
Source: Front Cell Infect Microbiol. 2022 May 4;12:859439. doi: 10.3389/fcimb.2022.859439 (PMC9114767; doi:10.3389/fcimb.2022.859439)
Supplement: Supplementary Figure 1 — Confusion matrix of MIC categories for C. albicans and C. glabrata. (A) Comparison of YO versus MNV using breakpoints for C. albicans, (B) Comparison of YO versus MNV using ECV/ECOFF for C. albicans, (C) Comparison of YO versus MNV using breakpoints for C. glabrata, (D) Comparison of YO versus MNV using ECV/ECOFF for C. glabrata. VME: Very Major Error in red, ME: Major Error in orange, mE: Minor error in yellow, no errors in green and no analysis (NA) in grey. For each category of discrepancies, the number of event is indicated (n = xx) with the percent of possible comparisons it represents. [file Presentation_1.pptx]

## Slide 1
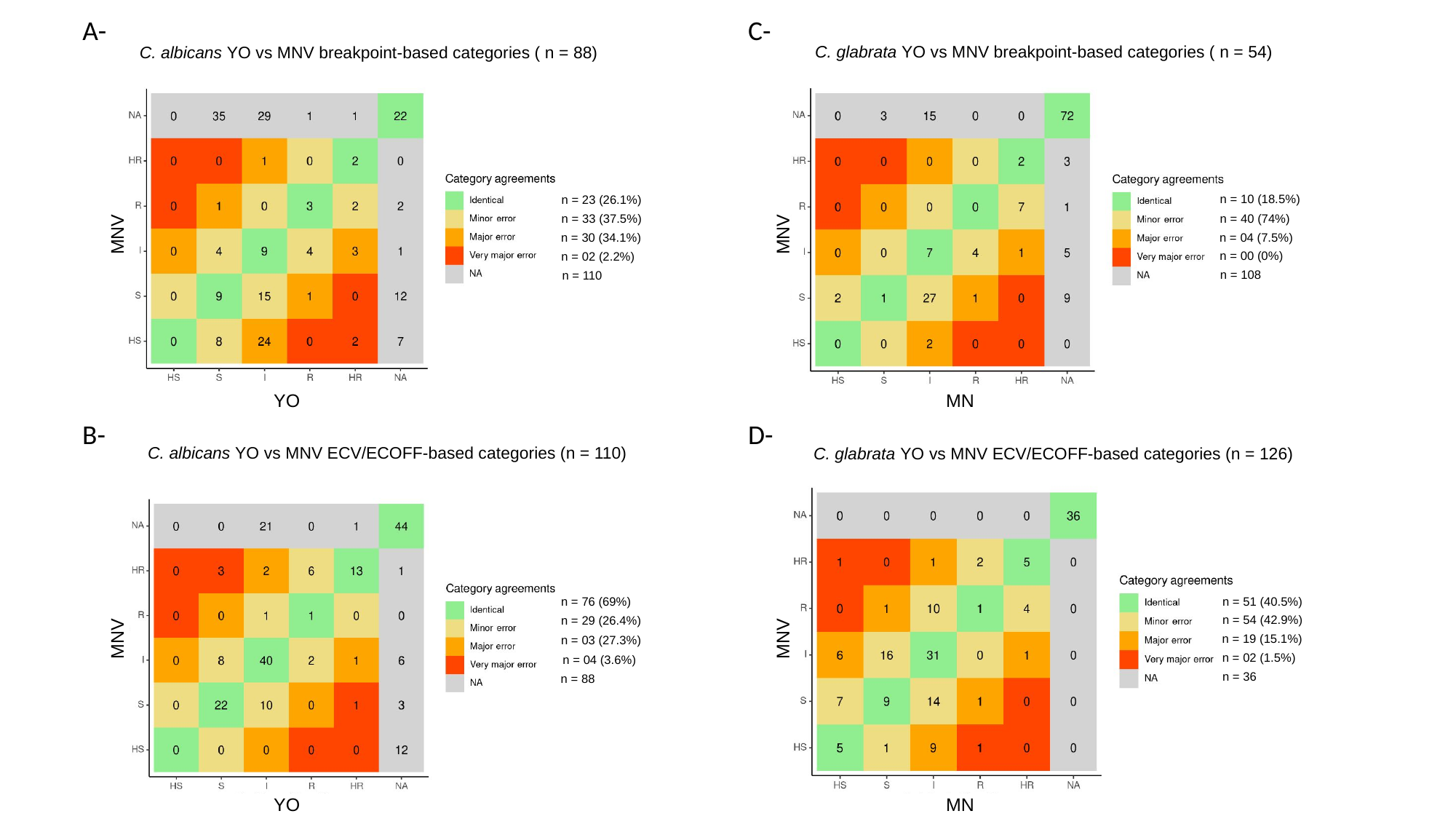

A-
C. albicans YO vs MNV breakpoint-based categories ( n = 88)
MNV
YO
C-
C. glabrata YO vs MNV breakpoint-based categories ( n = 54)
n = 10 (18.5%)
n = 23 (26.1%)
n = 40 (74%)
n = 33 (37.5%)
MNV
n = 04 (7.5%)
n = 30 (34.1%)
n = 00 (0%)
n = 02 (2.2%)
n = 108
n = 110
MN
B-
C. albicans YO vs MNV ECV/ECOFF-based categories (n = 110)
MNV
YO
D-
MNV
MN
C. glabrata YO vs MNV ECV/ECOFF-based categories (n = 126)
n = 51 (40.5%)
n = 76 (69%)
n = 54 (42.9%)
n = 29 (26.4%)
n = 19 (15.1%)
n = 03 (27.3%)
n = 02 (1.5%)
n = 04 (3.6%)
n = 36
n = 88
