# Supplementary material for: How Yeast Antifungal Resistance Gene Analysis Is Essential to Validate Antifungal Susceptibility Testing Systems
Source: Front Cell Infect Microbiol. 2022 May 4;12:859439. doi: 10.3389/fcimb.2022.859439 (PMC9114767; doi:10.3389/fcimb.2022.859439)
Supplement: Supplementary Table 1 — List of the strains used in this study. [file Table_1.docx]

Table S1: Strains used in this study

| Strain code | *species* | Strain name | reference |
| --- | --- | --- | --- |
| ALB0 | *C. albicans* | DSY 2323 | (Coste et al. 2007) |
| ALB1 | *C. albicans* | DSY 2322 |  |
| ALB2 | *C. albicans* | DSY 2321 |  |
| ALB3 | *C. albicans* | DSY 731 | (Coste et al. 2007) |
| ALB4 | *C. albicans* | DSY 732 |  |
| ALB5 | *C. albicans* | DSY 735 |  |
| ALB6 | *C. albicans* | DSY 741 | (Posteraro et al. 2009) |
| ALB7 | *C. albicans* | DSY 347 | (Coste et al. 2009:20) |
| ALB8 | *C. albicans* | DSY 286 | (Sanglard et al. 1995) |
| ALB9 | *C. albicans* | DSY 288 |  |
| ALB10 | *C. albicans* | DSY 289 |  |
| ALB11 | *C. albicans* | DSY 294 | (Coste et al. 2004) |
| ALB12 | *C. albicans* | DSY 296 |  |
| ALB13 | *C. albicans* | LS01 | (Coste et al. 2020) |
| ALB14 | *C. albicans* | LS02 |  |
| ALB15 | *C. albicans* | BS02 |  |
| ALB16 | *C. albicans* | BS01 |  |
| ALB17 | *C. albicans* | SG01 |  |
| ALB18 | *C. albicans* | DSY 2285 | (Dunkel et al. 2008) |
| ALB19 | *C. albicans* | DSY 2284 |  |
| ALB20 | *C. albicans* | DSY 3534 | (White 1997) |
| ALB21 | *C. albicans* | DSY 3548 |  |
| AUR1 | *C. auris* | Israel III.1 | (Ben-Ami et al. 2017) |
| AUR2 | *C. auris* | Israel III.8 |  |
| AUR3 | *C. auris* | LMDM-1218 | (Theill et al. 2018) |
| AUR4 | *C. auris* | LMDM-1219 |  |
| AUR5 | *C. auris* | LMDM-1231 |  |
| DUB1 | *C. dubliniensis* | 1015072877 | This study |
| DUB2 | *C. dubliniensis* | 8620914792 |  |
| DUB3 | *C. dubliniensis* | 8600931887 |  |
| DUB4 | *C. dubliniensis* | 1015113778 |  |
| DUB5 | *C. dubliniensis* | 1015087277 |  |
| DUB6 | *C. dubliniensis* | 1014779951 |  |
| FAM | *C. famata* | 1014337441 |  |
| GLA1 | *C. glabrata* | GE01 | (Coste et al. 2020) |
| GLA2 | *C. glabrata* | LU02 |  |
| GLA3 | *C. glabrata* | LU04 |  |
| GLA4 | *C. glabrata* | LS03 |  |
| GLA5 | *C. glabrata* | LS04 |  |
| GLA6 | *C. glabrata* | LS05 |  |
| GLA7 | *C. glabrata* | LS06 |  |
| GLA8 | *C. glabrata* | DSY 486 | (Ferrari et al. 2009) |
| GLA9 | *C. glabrata* | DSY 489 |  |
| GLA10 | *C. glabrata* | DSY 738 |  |
| GLA11 | *C. glabrata* | DSY 739 |  |
| GLA12 | *C. glabrata* | DSY 2234 |  |
| GLA13 | *C. glabrata* | DSY 2235 |  |
| GLA14 | *C. glabrata* | DSY 2253 |  |
| GLA15 | *C. glabrata* | DSY 2254 |  |
| GLA16 | *C. glabrata* | DSY 562 |  |
| GLA17 | *C. glabrata* | DSY 565 |  |
| GLA18 | *C. glabrata* | BS03 | (Coste et al. 2020) |
| GUI1 | *C. guillermondii* | 1014799355 | This study |
| GUI2 | *C. guillermondii* | 8600914257 |  |
| GUI3 | *C. guillermondii* | 8621290196 |  |
| KEF1 | *C. kefyr* | 1014372008 |  |
| KEF2 | *C. kefyr* | 1015364194 |  |
| KEF3 | *C. kefyr* | 8601420970 |  |
| KEF4 | *C. kefyr* | 1014452380 |  |
| KRU1 | *C. krusei* | 1014264884 |  |
| KRU2 | *C. krusei* | 1015067200 |  |
| KRU3 | *C. krusei* | 1014962063 |  |
| KRU4 | *C. krusei* | 1014787116 |  |
| KRU5 | *C. krusei* | 1013675193 |  |
| KRU6 | *C. krusei* | 1015302103 |  |
| KRU7 | *C. krusei* | 1016242422 |  |
| KRU8 | *C. krusei* | 1016226019 |  |
| LUS1 | *C. lusitaniae* | DSY 4593 | (Kannan et al. 2019) |
| LUS2 | *C. lusitaniae* | DSY 4606 |  |
| LUS3 | *C. lusitaniae* | DSY 4661 |  |
| LUS4 | *C. lusitaniae* | DSY 4590 |  |
| LUS5 | *C. lusitaniae* | DSY 4662 |  |
| LUS6 | *C. lusitaniae* | 8600849438 | This study |
| LUS7 | *C. lusitaniae* | 8600870966 |  |
| LUS8 | *C. lusitaniae* | 1015293296 |  |
| ORT1 | *C. orthopsilosis* | 1013898462 |  |
| ORT2 | *C. orthopsilosis* | 1013917705 |  |
| ORT3 | *C. orthopsilosis* | 1016285481 |  |
| MET | *C. metapsilosis* | 1013898929 |  |
| NIV | *C. nivariensis* |  |  |
| PAP1 | *C. parapsilosis* | 1014365888 |  |
| PAP2 | *C. parapsilosis* | 1015112863 |  |
| PAP3 | *C. parapsilosis* | 8600787306 |  |
| PAP4 | *C. parapsilosis* | 1014371308 |  |
| PAP5 | *C. parapsilosis* | 1013898363 |  |
| PAP6 | *C. parapsilosis* | 8600801680 |  |
| PAP7 | *C. parapsilosis* | 1015544862 |  |
| TRO1 | *C. tropicalis* | 8601114656 |  |
| TRO2 | *C. tropicalis* | 1015302004 |  |
| TRO3 | *C. tropicalis* | 8611162204 |  |
| TRO4 | *C. tropicalis* | 1015562743 |  |
| TRO5 | *C. tropicalis* | 8611112480 |  |
| DENEO | *Cry. deneoformans* | 1014882330 |  |
| NEO | *Cry. neoformans* | 1014882330 |  |
| LAU | *Cry. laurentii* | 1015251500 |  |
| CER | *S. cerevisae* | 1014264884 |  |
| CLA | *S. clavata* | 1011508943 |  |
